# Low-Grade Serous Carcinoma of the Ovary: The Current Status

**DOI:** 10.3390/diagnostics12020458

**Published:** 2022-02-10

**Authors:** Abdulaziz Babaier, Hanan Mal, Waleed Alselwi, Prafull Ghatage

**Affiliations:** 1 Department of Gynecologic Oncology, King Fahad Specialist Hospital, Dammam 32253, Saudi Arabia; 2 Department of Gynecologic Oncology, Tom Baker Cancer Centre, Calgary, AB T2N 4N2, Canada; hanan.mal@gmail.com (H.M.); prafull.ghatage@albertahealthservices.ca (P.G.); 3 Department of Adult Medical Oncology, King Fahad Specialist Hospital, Dammam 32253, Saudi Arabia; drwaleedalselwi@yahoo.com

**Keywords:** low-grade serous ovarian carcinoma, grading system, molecular profile, prognostic factors, cytoreductive surgery, targeted therapy

## Abstract

Low-grade serous carcinoma (LGSC) of the ovary is a rare histological subtype of epithelial ovarian carcinoma. It has distinct clinical behavior and a specific molecular profile. Compared with high-grade serous carcinoma, this tumor presents at a younger age, has an indolent course, and is associated with prolonged survival. LGSC can arise de novo or originate following a serous borderline tumor (SBT). Pathological differentiation between LGSC and other ovarian carcinoma histological subtypes is fundamental. Several factors might influence the overall outcome, such as the age at diagnosis, current smoking, elevated body mass index, mutational status, hormonal receptors’ expression, and Ki-67 proliferation index. Surgery is the main treatment option in LGSC, and efforts must be maximized to achieve a microscopic residual in metastatic disease. Despite being relatively chemo-resistant, adjuvant platinum-based chemotherapy remains the standard of care in LGSC. Hormonal maintenance therapy after adjuvant chemotherapy results in improved outcomes. Treatment options for disease recurrence include secondary cytoreductive surgery, chemotherapy, hormonal therapy, targeted therapy, and clinical trials. Advancements in genomic studies and targeted therapies are expected to change the treatment landscape in LGSC.

## 1. Background

Ovarian cancer remains the most lethal gynecologic malignancy [1]. Epithelial ovarian carcinoma (EOC) is the most frequent histological subtype. Based on histopathology, immunohistochemistry, and molecular analysis, EOCs are divided into five main subtypes: high-grade serous carcinomas (HGSC), endometrioid carcinomas, clear-cell carcinomas, mucinous carcinomas, and low-grade serous carcinomas (LGSC) [2]. HGSC is the most common ovarian epithelial carcinoma, while LGSC is rare. LGSC represents 2–5% of ovarian carcinomas and 5–10% of serous ovarian carcinomas [3,4]. The low prevalence of this disease results in limited data on the disease distribution, specific factors that impact the outcome, and descriptions of patients’ experiences. 

Although historically, it was thought that high-grade and low-grade serous tumors existed in a continuum, it has become clear they are two separate entities. They progress through independent pathways and behave differently in their clinical course, and ultimately, in their overall prognosis. LGSC has a distinct clinical behavior and a unique molecular landscape. LGSC is characterized by a younger age at diagnosis, indolent progression, relative chemo-resistance, and long-term survival. The average age at diagnosis is 55.5 years, compared to 62.6 years in HGSC [5]. Other reports even documented a younger median age at diagnosis of 45 years. The disease might affect women as young as 19 years old and as old as 79 years old [6]. The stage distribution of LGSC is similar to HGSC, with 80% of the patients diagnosed at an advanced stage [7]. Despite an equal stage distribution, the survival rate for patients with LGSC is superior (Table 1).

Research identifying the risk factors for developing LGSC has been limited due to the paucity of the disease. Current or previous history of a serous borderline tumor (SBT) of the ovary increases the risk of LGSC. The overall recurrence rate for previously treated SBTs is about 11%, and the absolute rate for malignant transformation of these patients is 2–4% [8]. Longacre et al., in a review of 276 cases, suggested that the potential malignancy risk is up to 6.9% [9]. However, when SBTs with peritoneal implants recur, most are LGSC. Crispen et al. evaluated 53 patients with progressive or recurrent SBTs of the ovary. They observed that 73% of the relapses contained LGSC elements [10]. Although germline *BRCA* mutations occur in a relatively high proportion of women with HGSC, LGSC does not appear to be part of the hereditary breast-ovarian cancer syndrome. Patients with LGSC are less likely to have a first- or second-degree relative with ovarian cancer [11]. Furthermore, an elevated body mass index (BMI) may increase the risk of LGSC. That could be explained by finding a high number of Müllerian inclusion cysts in the ovary due to the increased level of estrogen and androgens, which could be the origin of low-grade ovarian neoplasm [12]. 

Patients with LGSC usually have an indolent clinical course. Nevertheless, they experience multiple recurrences and may eventually die from disease progression [13]. 

## 2. Tumorigenesis/Histogenesis

LGSC can arise de novo or originate following a serous borderline tumor. The pathogenesis begins from a serous cystadenoma or adenofibroma in a sequential and indolent manner, which then develops into a SBT with either invasive or noninvasive implants, and finally reaches its full pathologic potential in the form of LGSC. Although the pathologic progression is thought to be sequential, parts of the sequence are occasionally omitted, and LGSC may arise directly from a classic SBT [14]. Several explanations support the stepwise progression theory:

(1) 60% of LGSCs contain areas of SBT [7].
(2) The potential progression risk of a SBT to LGSC is about 7% [10].
(3) A SBT that presents at an advanced stage tends to recur as LGSC [7].
(4) There are resemblances in the genomic profiles of SBT and LGSCs [15].
(5) Both types of ovarian SBT, classic and micropapillary/cribriform, have numerous allelic imbalances on multiple chromosomal arms (1 p, 5 q, 8 p, 18 q, 22 q, and Xp), which are also common in LGSC. The number of allelic imbalances progressively increases from SBT to LGSC [15].

However, there has been controversy around the cells of origin of LGSC. Some investigators have suggested the fallopian tube as the origin of LGSC [14,16,17]. It is postulated that most epithelial inclusion glands are tubal in origin rather than ovarian due to invagination of the ovarian surface epithelium with metaplasia. The fallopian tubal epithelia at the fimbriated end adhere to the ovarian surface. This process is facilitated by chronic inflammation, ovulation, and nonovulation-induced disruption of the ovarian surface. The adherent tubal epithelia then have a chance of invagination into the ovarian cortex to form ovarian epithelial inclusions, which are potentially the origin of serous cystadenoma, SBT, and then LGSC [16,17]. Furthermore, Vang et al. proposed a tubal pathway of pathogenesis for SBT and LGSC. They hypothesized that papillary tubal hyperplasia (PTH) is the origin of the SBT, noninvasive implant, and endosalpingiosis. Detached papillary tufts and epithelial clusters of PTH may exfoliate and implant on the ovary and peritoneum to form any of these lesions. Alternatively, a non-PTH tubal pathway may also evolve these lesions, whereby the normal tubal epithelium may exfoliate and implant on the peritoneum or the ovary [17].

## 3. Molecular Profile

Interest in molecular studies in cancer management has substantially increased in recent years. It is believed that exploring the molecular profile of a specific cancer should enhance the understanding of its clinical behavior and aid in developing an active targeted therapy. LGSC has a molecular landscape that is distinct from those of all other EOCs, including HGSC. 

A characteristic feature of LGSC is activating the mitogen-activated protein kinase (MAPK) pathway. It is a main intracellular signaling pathway that regulates essential cellular activities such as cellular proliferation, survival, migration, and angiogenesis. It mediates the transmission of growth signals into the nucleus. Mutations of either *BRAF* or *KRAS*, the upstream regulators of the MAPK pathway, result in constitutive activation of MAPK, which consequently activates a variety of cellular and nuclear targets downstream [18]. Singer et al. evaluated the incidence of *KRAS/BRAF* mutations in the LGSC of the ovary. They found that *BRAF* and *KRAS* mutations occurred in 33% and 35% of cases, respectively, though none of the tumors contained a mutation of both *BRAF* and *KRAS*. In contrast, none of the HGSCs harbored either a *BRAF* or a *KRAS* mutation [19]. Subsequently, other investigators have assessed the occurrence of these mutations in LGSC as well. Most of them reported a lesser proportion of these mutations. The percentage for *BRAF* mutations ranged between 2–10%, while the range for *KRAS* mutations was 19–40% [20,21,22,23,24,25]. In addition, Emmanuel et al. identified that 9% of LGSCs had *NRAS* mutations [26]. This mutation was exclusive to LGSC and did not exist in borderline tumors. The authors conclude that *NRAS* could be an oncogenic driver in serous carcinomas.

Another overexpressed pathway in LGSC is the insulin-like growth factor (IGF) pathway. Current evidence indicates that the IGF–insulin pathway may play a central role in ovarian carcinogenesis. IGF-1 is frequently overexpressed in LGSC compared with SBT and HGSC. The union of IGF with its receptor (IGFR) activates downstream effectors such as PI3K/AKT/mTOR, RAF, and MAP, which have been related to the pathogenesis of these tumors. Activating mutations of *PIK3CA* are detected in 40% of tumors, while inactivating *PTEN* mutations are present in 3–8% of tumors [27,28].

Cheasley et al. recently published the largest genetic study of LGSC of the ovary. They presented a comprehensive genetic analysis of 71 cases. It was reported that 90% of LGSC of the ovary harbored at least one potentially actionable alteration. Mutually exclusive mutations in RAS/RAF signaling dominated the mutation profile at 46.5%, made up of the following: *KRAS* 26.7%, *BRAF* 12.6%, and *NRAS* 8.5%. Ubiquitin-specific protease 9X (*USP9X*) is an X-linked deubiquitinase that plays a key role in tissue homeostasis. The *USP9X* somatic mutation was found in 15.5% of cases and copy number loss in 11.2%. Collectively, the frequency of cases with the *USP9X* mutation was comparable to those with the *KRAS* mutation. Known driver genes were identified as follows: *MACF1* (11.2%), *ARID1A* (9.9%), *NF2* (4.2%), *DOT1L* (5.6%), and *ASH1L* (4.2%). CDKN2 recurrent loss of expression or overexpression were registered as 9.9% and 5.6%, respectively [29]. 

It is understood that serous carcinomas follow a dualistic pathway of development. Low-grade tumors harbor mutations in the MAPK pathway, and high-grade serous carcinomas are universally characterized by *TP53* mutations. Serous carcinomas are divided into LGSCs and HGSCs, as separate histotypes and not only a graded continuum [15,19]. Table 2 summarizes the molecular feature of LGSC in comparison to HGSC.

## 4. Pathological Aspects

### 4.1. Grading System for Serous Carcinoma

Traditionally, serous carcinomas of the ovary were categorized according to three three-tier grading systems. These systems were the FIGO (the International Federation of Gynecology and Obstetrics) system, the World Health Organization (WHO) system, and the Shimizu/Silverberg system. The FIGO system evaluated the architectural features of the tumor [30], while the WHO system was based on architectural and cytologic features [31] and the Shimizu/Silverberg system examined three parameters: the glandular architecture, degree of nuclear atypia, and mitotic index [32]. Based on these systems, serous carcinoma would be classified as grade one, two, or three.

In 2004, Malpica et al. defined a novel two-tier system for grading serous ovarian carcinoma into a high grade or low grade [14]. The system is based primarily on the degree of nuclear atypia and uses the mitotic rate as a secondary criterion. In the binary system, tumors with mild to moderate nuclear atypia and a mitotic index of up to 12 mitoses per 10 high-powered fields are classified as low-grade serous cancers. In contrast, tumors with marked nuclear atypia and a mitotic index of >12 mitoses per 10 high-powered fields are classified as high-grade serous cancers. Since its introduction, the system has been validated, allowing its widespread use. The two-tier grading system has proven to have good inter-observer and intra-observer reproducibility [14,33]. Moreover, Bodurka et al. confirmed the superiority of the binary system in predicting clinical outcomes [34]. This user-friendly system has shown a good concordance with other grading systems, with the added advantage of having only two categories [7]. Replacing the historical three-tier systems with the binary system has standardized the diagnosis of LGSC internationally and inspired the medical community to genuinely investigate the difference between HGSC and LGSC in terms of molecular biology and clinical behavior.

### 4.2. Gross Features

LGSC of the ovary is bilateral in >50% of the cases. The size might range from 1 cm to more than 20 cm [6,35]. It is usually multicystic, involving the ovarian surface and parenchyma with papillary excrescences. The contents are serosanguinous or watery, straw-colored, and less frequently mucinous [6].

### 4.3. Microscopic Features

Accurate pathological evaluation is crucial to managing LGSC properly. It typically shows uniform cells with frank destructive invasion associated with mild to moderate atypia and a low mitotic index. 

**Figure 1:**
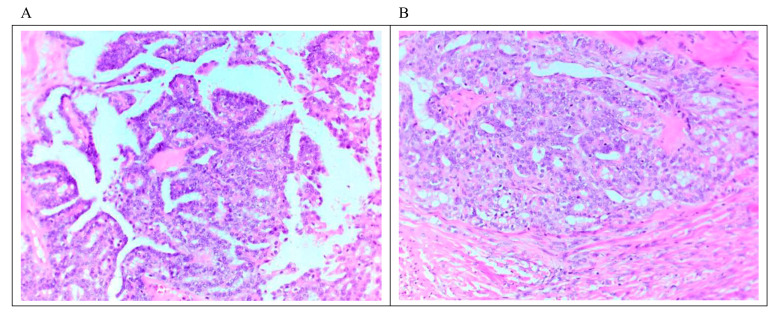
Microscopic feature of LGSC. (**A**) Micropapillary architecture outlined by cuboidal cells with mild to moderate atypia associated with low mitosis. (**B**) Focus on stromal invasion by the tumor with a cribriform architectural pattern. Scale bar 10 μm (20 × magnification).

LGSC is characterized by a monotonous population of cuboidal, low columnar, and sometimes flattened cells with an amphophilic or lightly eosinophilic cytoplasm. The degree of atypia is mild to moderate, with evenly distributed chromatin; occasional cells with larger nuclei can be seen. As mentioned above, the number of mitoses is no more than 12 mitoses per 10 high-power fields. Destructive invasion is recognized by neoplastic cells in the tumor/ovarian stroma in an area that measures ≥3.0 mm in linear dimension or has desmoplasia. The invasive component may grow in various architectural patterns such as micropapillary, cribriform, elongated papillae, glandular, medium-sized papillae, nests, macro-papillae, cell clusters, and as single cells (Figure 1). Commonly, there is a mix of architectural patterns. Psammoma bodies are common in LGSC and may be numerous. Occasionally, extracellular and intracellular mucin can be observed. Necrosis or multinucleated tumor giant cells are not commonly seen [6,7,35,36]. Sporadically, LGSC is associated with high-grade serous carcinoma at presentation or recurrences [37].

LGSC may primarily originate from the peritoneum rather than the ovary. Primary peritoneal carcinoma (PPC) is diagnosed based on the gynecology oncology group (GOG) criteria. The ovarian component must be nonexistent, confined to the surface with no cortical invasion, or involving the ovarian surface and underlying cortical stroma without any focus on the stroma, measuring >5 mm in depth and width [38]. Once the criteria for LGSC and PPC are met, the diagnosis of LGSC of the peritoneum is made.

Psammocarcinoma is a rare variant of serous neoplasm arising from the ovary or peritoneum that is separate from LGSC. It is described as a serous neoplasm exhibiting the following pathological features: (a) destructive invasion, (b) no more than moderate atypia, (c) no areas of solid epithelial proliferation except for occasional nests no more than 15 cells in diameter, and (d) at least 75% of papillae or nests associated with or entirely replaced by psammoma bodies. Psammocarcinoma has limited malignant potential and a favorable prognosis [39,40].

### 4.4. Immunohistochemical (IHC) Profile

IHC analysis has become an integral part of the pathological assessment. Therefore, understanding the staining pattern of LGSC should facilitate reaching a definite diagnosis. LGSC is usually stained positively for Wilson tumor-1 protein (WT-1), a marker used to confirm serous differentiation, and PAX-8, an indicator used to confirm a Müllerian origin [41]. In addition, most cases express estrogen receptors (ER), and some express progesterone receptors (PR) and E- cadherin. PAX-2 is overexpressed in 67% of the SBT and 50% of LGSC but never expressed in HGSC [42]. 

Her-2/neu expression is detected in about 28% of the patients and c-kit is positive in 4.5% of the cases [43]. Furthermore, P16 expression is mainly patchy or focal but sometimes can be diffuse or strong [44]. P53 expression is mostly wild-type but it might be overexpressed in 18% of the cases [41,45]. Rare cases of LGSC with the *BRAF* mutation are positive for VE1 (*BRAF* V600E) protein expression [46]. The Ki-67 proliferative index is typically positive in less than 10% of the tumor cells, while a higher index can also be seen [41,44]. Table 3 summarizes the typical LGSC IHC profile in relation to HGSC.

## 5. Investigation 

The clinical presentation of LGSC of the ovary is comparable to HGSC, including early satiety, vague abdominal or pelvic pain, abdominal bloating or distention, and altered bowel or urinary function. Patients may also present with bowel obstruction or pleural effusion. As mentioned above, most patients present at an advanced stage. Compared to HGSC, patients with LGSC tend to be younger, have a higher BMI, and are less likely to have ascites [47].

The diagnostic workup typically involves a thorough history and physical examination including pelvic examination, obtaining tumor markers (CA125), and proceeding with imaging modalities such as an abdominal and pelvic ultrasound and computed tomography (CT) of the chest, abdomen, and pelvis. The differential diagnosis of LGSC includes teratoma, Brenner tumor, SBT, HGSC, endometroid adenocarcinoma, and clear-cell carcinoma.

### 5.1. Tumor Marker (CA-125)

CA-125 (cancer antigen 125) is a protein encoded by the *MUC16* gene in humans. The serum tumor marker is most closely associated with EOC. Elevations in serum CA-125 values (>35 U/mL) have been recognized in over 85% of women diagnosed with ovarian cancer, specifically in those with advanced-stage disease [48]. Its level has proven valuable for diagnosing EOC, describing disease prognosis, and monitoring treatment [49]. 

In an ancillary analysis of GOG 182, Fader et al. reported the prognostic significance of CA-125 level in LGSC of the ovary. They showed that LGSCs have a significantly lower median pretreatment CA-125 value, and that overall, there are fewer patients with an elevated level when compared with HGSCs. It is probable that the HGSCs produce more CA-125 antigen on the surface of the ovarian cancer tumor cells or that the lower mitotic index of the LGSCs may lead to fewer antigens shed into the bloodstream. Pretreatment CA-125 did not correlate with the outcome. Yet, patients with CA-125 levels that normalized after the first, second, or third cycle were 60% less likely to develop disease progression when compared to those who never normalized or normalized after four cycles. The earlier normalization of CA125 levels was associated with a decreased risk of death. The adjusted hazard ratios (HR) for death from disease in patients that normalized after one, two, or three cycles were 0.45 (*p* = 0.025), 0.65 (*p* = 0.24), and 0.42 (*p* = 0.06), respectively. Furthermore, the median overall survival (OS) for those who did not experience normalization of CA-125 was 23 months, compared with 77.7, 46.8, 53.4, and 38.1 months for those who normalized before cycles two, three, four, and five, respectively [47]. Hence, CA-125 may be a sensitive biomarker of response to treatment and is predictive of the outcome in LGSCs.

Conversely, Schmeler et al. evaluated 24 patients with LGSC who received neoadjuvant chemotherapy. Where 50% of the patients had a reduction of 50% or more in their CA-125 level after neoadjuvant chemotherapy, only one (4%) had an objective response on imaging studies, though the majority of patients had stable disease [50]. The fall of the CA-25 level despite the absence of a clinical response can be explained by desmoplasia, calcification, and fibrosis, which are usually associated with LGSCs. It seems CA-125 has limited utility as a biomarker to predict the clinical response in the neoadjuvant setting.

### 5.2. Ultrasonography

A pelvic ultrasound is the first radiological modality utilized to characterize adnexal and ovarian lesions. Certain ultrasonographic features are correlated with malignancy. These features are thick irregular walls over 3 mm, papillary projections, and solid echogenic nodules with high vascularity on a color Doppler study [51]. 

LGSC usually appears as a multilocular cystic lesion with a higher number of solid components when compared to a SBT and with fewer solid components to HGSC. Calcifications are commonly appreciated secondary to the presence of psammoma bodies. Nevertheless, HGSC naturally tends to show a non-papillary solid mass with areas of cystic change, necrosis, and hemorrhage. A Doppler ultrasound can also be beneficial; in this case, HGSC tends to be more vascular than LGSC and SBTs [51,52].

### 5.3. Computed Tomography (CT) Scan

Contrast-enhanced CT continues to be the imaging modality of choice for the assessment of metastatic disease, with the ability to evaluate and detect lymphadenopathies and peritoneal metastases with a diagnostic accuracy of up to 89%. Oral and intravenous contrast is recommended to detect adnexal lesions and distinguish peritoneal metastases from adjacent structures. The sensitivity of CT to detect peritoneal metastases depends on their size; it is about 25–50% sensitive for lesions of less than 1 cm [51,53]. LGSCs appear as large, complex cystic lesions with well-circumscribed septa, papillary projections, and solid components. The tumor can either be unilateral or bilateral. The presence of calcifications in the adnexal mass or peritoneal metastasis is frequent in these lesions. Therefore, LGSCs must be differentiated from adnexal masses that also show calcifications, such as leiomyomas, Brenner tumors, fibromas, and teratomas. Nodal calcification, peritoneal implants, papillary projections in a cystic lesion, and necrosis in a solid mass are suggestive of malignancy and help distinguish LGSCs from benign masses [54].

### 5.4. Magnetic Resonance Imaging (MRI)

MRI is an imaging modality that lacks radiation exposure and enables superior soft-tissue characterization. It helps in verifying large adnexal masses detected on an ultrasound or CT, with high sensitivity (83%), specificity (84%), and diagnostic accuracy (83%). Features of malignancy in MRI are like those seen on an ultrasound or CT. The tumor may show early enhancement in dynamic contrast-enhanced MRI and high signal intensity in diffusion-weighted MR images [51,52].

Kawaguchi et al. reported on the MRI findings of LGSCs compared to SBTs. The signal intensities of solid components on T2-weighted images and the apparent diffusion coefficient (ADC) values were significantly lower in LGSCs than in SBTs [55]. Additionally, the ADC values of LGSCs in their study were higher than those of HGSCs in a previous report [56].

### 5.5. Positron Emission Tomography (PET) Scan

Fluorodeoxyglucose positron emission tomography (FDG PET) has limited value for the initial assessment of ovarian cancer. The physiological increased FDG uptake on the bowel loops and possible benign ovarian lesions might result in false-positive findings [51]. There is no correspondence between the ovarian cancer histological grade and the degree of FDG uptake. LGSCs might show greater FDG uptake than some HGSCs [57]. 

The PET scan plays a considerable role in diagnosing disease recurrence. Takeuchi et al. looked at the utility of PET/CT scans in LGSC. Thirty percent of PET/CT scans influenced the treatment course. The sensitivity, specificity, and accuracy of PET/CT were 94%, 100%, and 97%, respectively. Table 4 summarizes the performance of PET/CT in detecting recurrence of LGSC compared to CT scans and CA-125 (61). There is no statistically significant difference in sensitivity between PET/CT and CT (*p* = 0.13).

## 6. Prognostic Consideration

### 6.1. Clinico-Demographic Factors 

Several patient-specific factors have been explored and could contribute to the survival outcomes in women with LGSC. The age at diagnosis, smoking, and elevated BMI could impact the prognosis. Gershenson et al. analyzed the impact of age on the outcomes in LGSC [59]. Survival outcomes differ by age, and younger patients have a worse prognosis. Women diagnosed at age above 35 years have a significantly better progression-free survival (PFS) than younger patients (32.6 months vs. 18.8 months). The likelihood of progression or recurrence is lower in women over 35 years (HR 0.55) than women aged 35 years or less. The risk of death in females above 35 years is just under half of that compared to females who are 35 years or younger at the time of diagnosis (HR 0.53). Furthermore, the primary disease site impacts the outcome as well. Women with LGSC of the peritoneum have a statistically significant longer PFS and overall survival (OS) compared to LGSC of the ovary (36.2 vs. 25.4 months and 129 vs. 95.2 months, respectively).

Smoking negatively affects survival outcomes in women with LGSC [60]. Current smoking is significantly associated with a higher likelihood of dying (HR 2.08). Moreover, it is associated with statistically significantly shorter survival. The median OS is 79.9 months for never/former smokers vs. 48 months for current smokers. Current smokers are diagnosed with LGSC significantly younger than never/former smokers (mean age, 37 years vs. 45.1 years). Moreover, the BMI independently correlates with survival outcomes [60]. Schlumbrecht et al. reported that a BMI ≥ 35 kg/m^2^ is significantly associated with an increased likelihood of death (HR 2.53 [95% CI, 1.19–5.38]). That could be attributed to the technical challenges associated with an elevated BMI, leading to incomplete cytoreductive surgeries.

### 6.2. Mutational Status

The mitogen-activated protein kinase (MAPK) cascade plays a prominent role in the pathogenesis of LGSC. Mutations in *BRAF* and *KRAS* are frequent in SBT and low-grade invasive carcinomas [20,21,22,23,24,25]. The prognostic influence of these mutations was evaluated in several studies. Gershenson et al. analyzed the impact of a *BRAF/KRAS* mutation on the survival in 79 cases of LGSC [24]. The presence of either mutation was associated with a significantly better OS than those with wild-type *BRAF* or *KRAS* (106.7 vs. 66.8 months). A *KRAS/BRAF* mutation showed a protective effect on OS (HR 0.49). In patients with SBTs, *BRAF* mutations are more common in the early stage than in the advanced stage. Tsang et al. assessed the frequency of *BRAF* and *KRAS* mutations in 23 patients with advanced-stage SBTs who developed a recurrence of LGSC and 13 patients with advanced-stage borderline tumors and no evidence of disease progression. Only 1 of 23 patients with SBTs and a recurrence of LGSC had a *BRAF* mutation (4.3%), whereas 18 of 23 patients had a *KRAS* mutation (78.3%). These data may indicate that advanced-stage LGSC is more likely to be derived from SBTs without *BRAF* mutations. Furthermore, a *BRAF* mutation in a SBT may protect the individual from developing a subsequent LGSC [61]. Likewise, Grisham et al. concluded that a *BRAF* mutation is associated with an early stage at presentation, borderline serous histology, and improved outcome. Yet, they proposed that the presence of a *BRAF* mutation in patients with borderline serous disease precludes progression to more aggressive disease [23].

Interestingly, Tsang et al. reported shorter survival in patients with *KRAS G12V* mutations than those with *KRAS G12D*, wild-type, or rare *KRAS* variants [61]. The median survival times of patients with *KRAS G12V, KRAS G12D*, and wild-type *KRAS* or rare *KRAS* variants were 125, 189, and 168 months, respectively (HR 4.77).

### 6.3. Hormone Receptor (HR) Status

Expression of estrogen receptors (ER) and progesterone receptors (PR) has been documented in all histological types of ovarian carcinoma [62]. Voutsadakis published a meta-analysis of the hormone receptors’ expression in LGSC [63]. When analyzing nine studies including 437 patients with LGSC, the ER expression was 80.7% (72.2–89.1%). Among seven studies including 374 patients, the PR expression was estimated as 54.4 % (44.3–64.4%). 

Fernandez et al. looked at the predictive value of the hormone receptors in 55 patients with advanced LGSC [64]. Both ER and PR Allred expression scores were significantly associated with OS outcomes in univariate analysis. Remarkably, ER expression had no significant effect on PFS, while the PR Allred score did. Using the Allred scoring system, LGSC tumors with higher levels of ER expression by IHC (≥7) were significantly correlated with better OS independently of the PR expression, age, and residual disease. Conversely, low PR Allred scores showed inferior PFS.

Moreover, Sehouli et al. studied the prognostic implication of hormone receptors’ expression in 68 patients with LGSC. The percentage of HR expression proved to be a favorable prognostic indicator for PFS, although it only exhibited a tendency for improved OS. The statistically significant cutoff for PFS was 75% positive tumor cells for ER and 15% positive tumor cells for PR. There was a tendency for there to be a better OS for HR-positive tumors, but a statistically significant cutoff could not be determined [65].

### 6.4. Ki-67 Proliferation Index

Ki-67 is a marker in the nuclear matrix during cell division. The role of Ki-67 appears to prevent chromosomes from sticking together by binding at one end to the chromosome and repelling other chromosomes with the positively charged other end. Ki-67 is expressed only during the cell cycle’s active G1, S, and G2 phases [66]. 

In addition to assessing the prognostic impact of hormone receptors in LGSC, Sehouli et al. evaluated the prognostic significance of the Ki-67 proliferation rate. Cox regression analysis revealed a significant continuous decrease in OS for a higher proliferation rate. A Ki-67 cutoff of 6.28% is an important factor for a poor outcome (HR 1.07). The median OS is significantly inferior if the Ki-67 is over 6.28% than it is for a lower proliferation rate (66 months vs. 83 months). Even after multivariate analysis, the Ki-67 index remained an independent prognostic factor for LGSC [65].

Grabowski et al. assessed the correlation between Ki-67 and treatment outcomes in LGSC. Patients with a Ki-67 expression level of ≥3.6% had a significantly higher likelihood of residual disease. Ki-67 < 3.6% is associated with substantially longer therapy-free intervals. Additionally, Ki-67 ≥ 4.0% correlates with a superior response rate to chemotherapy [67]. Therefore, the Ki-67 index is a valuable tool as a prognostic factor, biomarker for therapy outcome, and complete resection predictor in LGSC.

## 7. Management

### 7.1. Primary Treatment 

#### 7.1.1. Surgical Management

Surgery is the management cornerstone of all EOCs, including LGSC. Surgical staging for apparent disease confined to the ovary includes total abdominal hysterectomy, bilateral salpingo-oophorectomy, pelvic and para-aortic lymphadenectomy, and omentectomy. However, in advanced disease, cytoreductive surgery might involve multiple resections to achieve optimal debulking.

Systemic lymphadenectomy is a routine procedure in the staging surgery of EOCs. The incidence of lymph node (LN) involvement in apparent early-stage LGSC is about 10%. Of note, 5% is upstaged based on positive LN alone [68]. Vatansever et al. retrospectively analyzed 191 patients with LGSC. Among 155 who underwent LN assessment, there were 44.5% overall with metastatic nodal disease, 2% with stage one, and 63% with advanced disease. Lymphovascular space invasion (LVSI) was strongly associated with LN involvement. If LVSI is present, the risk of LN metastasis is 68%, compared to 32% once the LVSI is negative. The five-year PFS for LVSI positivity was 31% and LVSI negativity was 74.7% [69].

In metastatic disease, effort should be directed toward maximum cytoreductive surgery to achieve microscopic residual. Several researchers have addressed the impact of residual disease on the prognosis of LGSCs. In an ancillary analysis of GOG 182, Fader et al. published findings on the survival of 189 patients with LGSC. Only the residual disease status was significantly associated with the survival. The median PFS for microscopic residuals, those less than 1 cm, and those over 1 cm were 32.2 months, 14.65 months, and 14.1 months, respectively. Again, the median OS for microscopic residuals, those less than 1 cm, and those over 1 cm were 96.6 months, 45 months, and 42 months, respectively. After controlling for other variables, patients with LGSC and measurable residual disease after primary cytoreductive surgery had an adjusted HR for disease progression of 2.28 and death HR of 2.12, which were comparable outcomes to HGSC with measurable disease [70]. Furthermore, Gershenson et al. concluded that having a measurable disease at the end of the primary cytoreductive surgery was associated with a 1.79 greater chance of progression or recurrence and 1.78 increased hazard of dying, compared with no visible disease at the end of the surgery [59]. Grabowski et al. performed an exploratory analysis of four phase-three clinical trials, and 145 patients with LGSC were evaluated. There were significant differences in OS and PFS between subgroups with microscopic residuals, with residuals between 1–10 mm, and residuals > 1 cm. The five-year OS in patients with tumor residuals > 1 cm was 32%, while 85% of women had complete cytoreduction The PFS and OS did not reveal any significant difference when the residual disease was equal to or more than 1 cm [71]. A recent retrospective analysis by Vatansever et al. for 191 patients with LGSC showed a similar impact of residual disease on the outcome [69]. All the above-cited studies confirmed that residual disease is the single important variable affecting the survival of patients with LGSC.

Fertility sparing surgery (FSS) can be offered to highly selected patients. Only women of reproductive age with stage 1A–C1 can have this conservative surgical option. Jiang et al. reported a retrospective analysis of 108 patients of reproductive age who were diagnosed with EOCs. They were treated either with FSS or standard surgery. The authors concluded that FSS was safe for patients of reproductive age with low-grade and stage-one conditions [72]. Fruscio et al. reported a long-term analysis of FSS in early-stage EOC. Their research included 1031 patients, of which 242 underwent FSS. Independent of the type of surgery, the outcome was inferior in women with grade-three conditions, and these patients had a risk of distant recurrence. However, they suggested that FSS can be offered to all young patients when the tumor is limited to the ovaries [73]. Finally, in a cohort study using the National Cancer Database, Melamed et al. identified 1726 women with stage 1A–C EOC, of whom 825 (47.8%) underwent FSS. The conservative approach was not associated with the hazard of death. They concluded that FSS was not associated with an increased risk of death in young women with stage-one EOC compared with radical surgery [74].

#### 7.1.2. Neoadjuvant Chemotherapy

If the likelihood of achieving optimal debulking is low or a patient’s medical condition does not allow major cytoreductive surgery, neoadjuvant chemotherapy (NACT) followed by interval debulking surgery (IDS) might be considered after histological confirmation of disease by tissue biopsy, not just by cytology. In two randomized clinical trials (RCT), such an approach proved non-inferior to primary cytoreductive surgery followed by adjuvant chemotherapy in advanced-stage EOCs. Most patients enrolled in these trials were of high-grade histology [75,76]. Schmeler et al. retrospectively reviewed the response rate to NACT in 25 patients with advanced LGSC. All patients received platinum-based chemotherapy and a median of six cycles. Most patients (88%) had stable disease (SD), one patient (4%) had a complete response (CR), no partial responses were identified, and two patients (8%) showed progression [50]. Subsequently, Cobb et al. assessed the role of NACT in LGSC compared to HGSC. They identified 36 patients with LGSC who received NACT and matched them with HGSC patients. Despite a significant reduction in CA-125 levels, the response rate in LGSC was significantly lower than in HGSC. Again, most patients showed stable disease (83%), four patients had partial responses (11%), and two patients (6%) revealed progression [77]. These findings indicate that LGSC is less responsive to NACT compared to HGSC. Hence, this approach should be considered with caution in LGSC.

#### 7.1.3. Adjuvant Chemotherapy

Adjuvant chemotherapy is recommended for all patients with LGSC not limited to the ovary. The 2020 National Comprehensive Cancer Network (NCCN) guideline does not recommend adjuvant therapy in stages 1A and 1B; then, while there is no treatment standard for those with stage 1C disease, observation, chemotherapy, or endocrine therapy are all recommended treatment options [78]. Even though LGSC is relatively chemo-resistant compared to HGSC, carboplatin and paclitaxel remain the standard of care in the adjuvant setting. Grabowski et al. reported a low response rate in LGSC to the standard chemotherapy. They analyzed 39 patients with a residual over 1 cm at the end of the debulking surgery. The complete or partial response was 23.1%, stable disease was 69.2%, and disease progression was 7.7% [71]. Gershenson et al. examined the clinical behavior of LGSC. Only 52% of 112 patients with LGSC were clinically disease-free at the end of primary platinum-based chemotherapy [79]. In comparison, around 80% of patients with HGSC showed no evidence of disease after platinum-based chemotherapy [80]. 

Angiogenesis has been shown to play a central role in the pathogenesis and clinical behavior of ovarian carcinoma. Bevacizumab, a monoclonal antibody directed against the vascular endothelial growth factor (VEGF), was evaluated in the first-line setting in the GOG 218 and ICON 7 clinical trials [81,82]. The addition of bevacizumab to the standard chemotherapy in advanced high-risk EOCs improved the PFS. Eighty patients with advanced LGSC were included in ICON 7. Adding bevacizumab resulted in a non-significant HR of 0.78 in this sub-analysis, backing the addition of bevacizumab. However, the trial was underpowered to detect any therapeutic effect of bevacizumab in LGSC [83].

#### 7.1.4. Hormonal Therapy

The low response rate to the standard chemotherapy and the overexpression of ER/PR receptors in LGSC have attracted investigators to explore the clinical value of hormonal therapy in LGSC. Gershenson et al. retrospectively investigated outcomes associated with hormonal maintenance therapy (HMT) in 203 patients with stage 2–4 LGSC [84]. They compared 70 patients who received HMT after primary cytoreductive surgery and chemotherapy with 133 who underwent observation alone. There was a significantly higher proportion of women in the HMT group with persistent tumors at the completion of primary chemotherapy (60.0% vs. 8.3%). The median PFS of patients who received HMT was 64.9 months vs. 26.4 months in the observation group. HMT was associated with a lower risk of progression compared with observation (HR 0.44). Patients with and without persistent disease at the end of platinum-based chemotherapy had a better PFS in the hormonal maintenance therapy group. HMT increased the median PFS by more than twofold. When the ER or PR expression status was analyzed, ER-positive or PR-positive patients who received HMT had considerably improved outcomes. There was no statistically significant difference in the OS between the two groups. Nevertheless, after adjusting for disease status, the median OS for the HMT group was significantly longer than that for the observation group.

Fader et al. published a retrospective analysis of 27 patients with stage 2–4 LGSC treated hormonally after cytoreductive surgery instead of with platinum-based chemotherapy. A microscopic residual was achieved in 85.2% of cases. ER-positivity was found in 96% of cases, as only 32% expressed PR. The reported three-year PFS was 79%, and the three-year OS was 92.6% [85].

### 7.2. Recurrence

Despite the relatively good prognosis of LGSC, over 80% of patients experience a relapse of disease. Mostly, these patients are young and are expected to handle aggressive therapy and potentially live for a long time. The treatment modalities in recurrence include secondary cytoreduction, chemotherapy, bevacizumab, hormonal therapies, targeted agents, and enrollment in clinical trials. There is no standard approach to treatment, but these options should be considered in every disease recurrence.

Secondary cytoreductive surgery (SCRS) requires careful patient selection. As with primary cytoreductive surgery, the main goal is to achieve microscopic residual disease. Several factors might influence the decision to proceed with another major debulking procedure. For instance, the patient’s age, performance status, time to recurrence, disease distribution, and ability to potentially remove all measurable disease. A retrospective study by Crane et al. of 41 patients with recurrent LGSC revealed that cytoreduction to microscopic residual disease could only be achieved in 22% of patients. The median PFS was statistically longer in patients with no residual tumor at the completion of surgery compared with those with a gross residual tumor (60.3 vs. 10.7 months). There was a trend toward longer OS for patients with no residual disease (167.5 vs. 88.9 months) but it did not reach a statistical significance [86]. The recently published DESKTOP III trial proved that SCRS followed by chemotherapy resulted in longer OS than chemotherapy alone. The microscopic residual resection rate was 74.2%. The median OS was statistically superior in the surgical arm (53.7 vs. 46 months). The OS benefit was highest among the complete resection cohort, with a median OS of 61.9 months compared to 46 months in the no-surgery arm. DESKTOP III was the first RCT to show a survival benefit from such an intervention in recurrent EOC. Of note, most enrolled patients had HGSC; only six patients with LGSC were involved in the analysis [87]. 

Rechallenging with chemotherapy is another choice to be considered. Gershenson et al. identified 58 patients with recurrent LGSC who received various chemotherapy regimens. The overall response rate was 3.7%, while in the platinum-sensitive cohort it was 4.9% and in the platinum-resistant cohort it was 2.1%. However, stable disease was observed in 60% of the patients [88]. Several regimens have been used in clinical practice, but pegylated liposomal doxorubicin (PLD) seems the most active regimen for recurrent LGSC. Rose et al. reported a complete response rate of 14.3%. The treatment of stable disease by PLD resulted in increased PFS in 78.6% of cases, by an average of 350% [89]. The addition of bevacizumab to chemotherapy for recurrent LGSC might enhance response rates. Dalton et al. reported a response rate of 47.5% for bevacizumab-containing regimens and 77.5% clinical benefit when stable disease was included in the analysis [90]. 

Hormonal treatment could represent an alternative option to chemotherapy for recurrent LGSC. Gershenson et al. retrospectively reviewed 64 patients treated with hormonal therapy in recurrent LGSC. The overall response rate was 9%, and stable disease was achieved in 61.8% of cases. The most common regimen was with an aromatase inhibitor. ER/PR expression data were available for 50 patients in this analysis. Patients with ER+/PR− tumors had a shorter time to progression (HR = 1.8) than patients with ER+/PR+ tumors. This observation approached but did not reach statistical significance [91]. In the PARAGON study, Tang et al. prospectively investigated the clinical benefit rate of anastrozole in patients with recurrent/metastatic LGSC and SBT, who were ER-positive and/or PR-positive. Thirty-six patients were enrolled, of whom 34 patients had LGSC. The best study response included a partial response in 14%, stable disease in 50%, and disease progression in 36% of cases. The authors concluded that anastrozole was associated with a clinical benefit rate of 61% for patients with recurrent ER-positive and/or PR positive LGSC for at least six months, with acceptable toxicity [92].

### 7.3. Hyperthermic Intraperitoneal Chemotherapy (HIPEC)

HIPEC incorporation in the management of metastatic ovarian carcinoma has been developing over the past two decades. In 2018, van Driel et al. confirmed a superior overall survival in patients who underwent interval debulking surgery (IDS) and received additional HIPEC. While this phase-three trial included all EOCs’ histological subtypes, only six patients with LGSC were involved in the analysis [93]. These exciting data resulted in more clinical trials exploring such an approach, with different regimens for managing EOCs. HIPEC has not become the standard of care yet, but enough evidence supports its integration in the management of EOC, including LGSC. The disease trajectory of LGSC involves multiple recurrences with relatively long survival, which necessitates several interventions. Therefore, exploring this treatment modality in the primary setting or disease recurrence seems sensible [94,95,96]. 

### 7.4. Targeted Therapy/Immunotherapy

When appreciating the limited efficacy of chemotherapy and hormonal therapy in managing LGSC, there is a strong rationale for exploring novel targeted agents that act against this indolent yet fatal disease. LGSC harbors several genes aberrations that activate the MAPK pathway. The MAPK cascade is prompted by the binding of ligands that eventually leads to phosphorylation of extracellular signal-regulated kinase (ERK). MEK is a major downstream protein in the MAPK pathway and thus an attractive target for inhibitor therapy. Various MEK inhibitors (MEKi) have been developed in the past decade [97]. In preclinical data on ovarian cancer, cell lines with *KRAS* or *BRAF* mutations experienced significant growth inhibition and apoptosis compared to wild-type cells [98].

In GOG 0239, Farley et al. assessed the safety and activity of selumetinib (MEKi) in recurrent LGSC. Fifty-two heavily pretreated patients were enrolled in this open-label single-arm phase-two study. The overall response rate was 15%, with predominantly partial responses. Stable disease was observed in 65% of cases. The median PFS was 11 months, with 63% of patients having a PFS over six months. Noticeably, there was no correlation between the tumor response and *KRAS* or *BRAF* mutational status. Selumetinib was associated with the well-described and acceptable toxicities of MEKi [22]. The findings suggested that inhibitors of the MAPK pathway deserve further research in LGSC. Additionally, the efficacy of trametinib, another MEKi, was evaluated in a randomized phase-two/three trial on recurrent or progressive LGSC. This trial (GOG 281) compared trametinib with the standard of care (one of five options: letrozole, tamoxifen, PLD, weekly paclitaxel, or topotecan). The median PFS was significantly improved with trametinib compared to the standard of care (13 vs. 7.2 months; HR 0.48; *p* < 0.0001). The overall response rate was statistically superior with trametinib (26.2% vs. 6.2%). Furthermore, the response duration for trametinib was significantly better than for the standard of care. Complete publication of the study is pending. In the interim, although MEK inhibitors are not currently US Food and Drug Administration approved for recurrent LGSC, trametinib was added to the NCCN ovarian cancer guidelines based on GOG 281 [99]. Subsequently, Monk et al. evaluated binimetinib, a potent MEK1/2 inhibitor, in recurrent or persistent LGSC. In a randomized trial (MILO/ENGOT-ov11), 303 patients were randomly assigned to binimetinib versus the physician’s choice of chemotherapy. The median PFS was 9.1 months for binimetinib and 10.6 months for the clinician’s choice of chemotherapy, resulting in early study closure for futility. The objective response rates, median duration of response, and median OS were similar between the groups. Post hoc analysis suggested a possible association between the *KRAS* mutation and response to binimetinib [100]. 

## 8. Conclusions/Future Directions

LGSC is a distinctive entity of EOC with a specific molecular alteration and patterns of clinical behavior. It accounts for <5% of ovarian carcinoma. LGSC used to be addressed like HGSC. However, patients with LGSC are usually diagnosed at a younger age, are less sensitive to the standard chemotherapy, have a longer disease trajectory, and experience fewer disease-free intervals. Thus, women with LGSC often repeatedly receive frequent treatment regimens.

Several factors might have implications for increasing the risk of developing LGSC. For instance, a previous history of SBT or obesity have been linked. Yet, the *BRCA* gene mutation does not seem typically associated with LGSC, and having a family history of ovarian cancer is less likely in these patients. LGSC grows either de novo or from a SBT, and the fallopian tube has been assumed to be the origin of LGSC cells. LGSC has characteristic microscopic features. It exhibits mild to moderate atypia with a mitotic index of up to 12 mitoses per 10 high-powered fields. Psammoma bodies are frequently seen, but necrosis is rare. IHC staining is not always indicated but could be helpful to confirm the diagnosis and rule out other possibilities.

LGSC may present as an early stage, or more frequently, as metastatic abdominal disease. CA-125 is part of the laboratory assessment of ovarian carcinoma. Generally, CA-125 in LGSC is lower than its level in HGSC. The CA-125 level is valuable for predicting the prognosis and monitoring the treatment response. Imaging modalities such as ultrasounds, CT scans, MRI, and PET scans are tools that have been used as part of the initial evaluation of LGSC or during surveillance. There are no classical radiological features, but some findings might suggest diagnosis of LGSC. Of them all, the PET scan has superior sensitivity, specificity, and accuracy in detecting disease recurrence.

Numerous factors influence the prognosis of LGSC. Women below the age of 35 have an inferior outcome, with a higher risk of progression and death from the disease. Patients with LGSC of the peritoneum have better PFS and OS than LGSC of the ovary. Current smoking and an elevated BMI are also associated with worse outcomes. In addition, the mutational status, hormone receptor expression, and Ki-67 proliferation affect the disease course. Mutation of *BRAF* or *KRAS* seems to have a protective effect on the OS. A *BRAF* mutation is believed to be associated with early disease and an improved outcome. However, a *KRAS G12D* mutation might correlate with disease progression and shorter survival. The hormone receptors of positive tumors showed longer PFS. Furthermore, a low Ki-67 proliferation index exhibited a superior prognosis.

Practice patterns may vary substantially due to a scarcity of randomized trial data to advise evidence-based treatment strategies. Nevertheless, surgery is the primary treatment option for LGSC, with comprehensive surgical staging for apparent early disease and cytoreductive surgery for metastatic disease. The size of residual disease directly influences survival. Therefore, cytoreductive surgery aims to remove all measurable disease and achieve a microscopic residual. The most important prognostic factor in the treatment of LGSC is optimal resection at the time of primary cytoreduction. The chance of attaining a microscopic residual in advanced LGSC is about 50–85% [69,70,71]. While LGSC is indolent and not as chemotherapy-sensitive as HGSC, it is not totally chemotherapy-resistant. Given the lack of a more proven, effective therapy, platinum-based adjuvant chemotherapy remains the standard of care for all LGSC with disease beyond the ovary. It is essential to highlight that there are no prospective randomized studies of first-line chemotherapy in women with LGSC. Maintenance hormonal therapy following adjuvant chemotherapy leads to a better outcome and should be considered after completion of the primary treatment. It is recommended that endocrine therapy, such as aromatase inhibitors or tamoxifen, is continued until disease progression or development of unacceptable toxicity. Once the disease recurs, the suitability for secondary cytoreductive surgery needs to be considered. As with the primary setting, debulking surgery aims to achieve no gross disease. Other options include rechallenging with chemotherapy or hormonal therapy. When the response to both therapies is low, enrollment in clinical trials should be considered. HIPEC can be regarded as a treatment option in the primary or recurrent setting, although data in LGSC are not well-established.

The limited efficacy of systemic chemotherapy and hormonal therapy necessitates that we address other treatment options, such as targeted therapy. LGSC is characterized by high mutational alterations in the MAPK cascade. Hence, inhibition of MEK potentially results in an anti-cancer effect. Investigations into selumetinib in a phase-two trial showed promising results with an acceptable toxicity profile [22]. Successively, several trials were initiated to investigate other MEKis in LGSC. In GOG 281, trametinib showed an improvement in PFS and the overall response rate compared with standard chemotherapy or hormonal therapy [99]. The result of this trial led trametinib to be included in the updated NCCN guideline for ovarian cancer. Several MEK inhibitors were evaluated in combination with other agents to enhance efficacy. Preclinical studies indicated that dual blockade of the MAPK and PI3K/AKT pathways was an effective treatment strategy in ovarian carcinoma. The results of a phase-two study of pimasertib (MEKi) with or without SAR245409 (PI3K/mTOR inhibitor) in women with previously treated unresectable LGSC or BST were disappointing. The study was terminated early because of the low response rate and high discontinuation. The MEK inhibitor alone was as effective as the combination. There was no statistically significant difference in response rate or PFS [101]. The MAPK signaling pathway’s complexity and role in LGSC are not fully understood. Vigorous translational research components within trials that aim to better outline the role of MEKi in LGSC are essential.

The insulin-like growth factor (IGF) pathway potentially presents an additional opportunity for developing a novel therapy. IGF-1 is overexpressed in LGSC. Downstream effectors of the IGF pathway include PI3K/AKT/mTOR and MAPK, which play a recognized role in LGSC tumorigenesis. Inhibition of this pathway could reveal anti-tumor activity. Furthermore, Huvila et al. recently described overexpression of the stimulator of interferon genes (STING) pathway in LGSC. STING is a part of the cGAS–STING pathway that mediates the innate immune defense against infectious DNA-containing pathogens. It also generates intrinsic anti-tumor immunity via tumor-derived DNA. The STING pathway is suppressed through epigenetic silencing or loss of function mutations. While these data require further validation, the STING pathway could offer a prospect of therapeutic intervention [102].

The recent publication by Cheasley et al. on the largest genomic evaluation of LGSC of the ovary should provide appealing targets for novel therapeutic agents [29]. Their analysis confirmed that about 90% of LGSC has at least one actionable alteration.

In January 2019, a group of basic, translational and clinical researchers and patient advocates assembled in Miami, Florida to discuss the current state of the science on LGSC. The aim was to introduce upcoming collaborations, laboratory studies, and clinical trials. to better comprehend this disease outside of the limits of single academic organizations. There is a pressing need for prospective randomized studies on LGSC. Novel trial designs are essential to studying rare cancers. Clinical trials to assess optimal therapeutic regimens in rare diseases have become costly and are problematic to complete due to the limited number of patients available for study. The Gynecologic Oncology Group (GOG) and the Gynecological Cancer Intergroup (GCIG) have created working committees focused on rare gynecological tumors, which help with implementing collaborations between institutions and overcoming obstacles with patient recruitment.

The emerging data suggest that a “one size fits all” approach to EOC treatment is no longer relevant. Therefore, there are multiple ongoing clinical trials (Table 5) to investigate several novel therapies in the management of LGSC. The data from these trials will change the treatment landscape for LGSC in the next few years.

## Figures and Tables

**Table 1:** Survival rate for LGSC compared to HGSC [5].

	LGSC	HGSC
Overall survival	99 months	57 months
Stage I	123 months	108 months
Stage II–IV	84 months	52 months
5-year survival	75%	40%
10-year survival	70%	26%
10-year survival rate stage I	92%	76%
10-year survival rate stage II–IV	55%	20%

LGSC: low-grade serous carcinoma; HGSC: high-grade serous carcinoma.

**Table 2:** Molecular landscape of LGSC vs. HGSC [15,19,27,28,29].

LGSC	HGSC
*KRAS* mutation *BRAF* mutation *NRAS* mutation *PIK3CA* mutation USP9X mutation IGF-1 overexpression PAX-2 expression Inactivation of *PTEN* mutation	*TP53* mutation *BRCA* 1& 2 mutation P16 expression *NF1* mutation *PB1* mutation *CDK12* mutation

**Table 3:** Typical IHC profile of LGSC compared to HGSC.

Biomarker	LGSC	HGSC
WT-1	Positive	Positive
PAX-8	Positive	Positive
ER	Mostly Positive	Mostly Positive
PR	Possibly Positive (~50%)	Possibly Positive (~30%)
MIB1	Mainly negative	Possibly positive (~50%)
E-cadherin	Possibly Positive	Mainly negative
PAX-2	Positive (~50%)	Negative
Her-2/neu	Possibly positive (~30%)	Possibly positive (~20%)
P16	Mainly patchy or Negative	Diffusely positive
P53	Mainly patchy or Negative	Diffusely positive (~90%)
Ki-67	Low	High

LGSC: low-grade serous carcinoma; HGSC: high-grade serous carcinoma; WT-1; Wilson tumor-1 protein; ER: estrogen receptor; PR: progesterone receptor.

**Table 4:** Performance of PET/CT in detecting recurrence of LGSCs compared to CT scan and serum CA-125 alone [58].

	PET/CT	CT	Serum CA-125
Sensitivity	94% (95% CI: 84–98%)	89% (95% CI: 78–96%)	68% (95% CI: 49–83%)
Specificity	100% (95% CI: 94–100%)	95% (95% CI: 88–99%)	89% (95% CI: 51–99%)
Accuracy	97% (95% CI: 93–99%)	93% (95% CI: 88–97%	73% (95% CI: 56–85%)

PET: positron emission tomography; CT: computed tomography; CI: confidence interval.

**Table 5:** Ongoing clinical trials in LGSC.

ClinicalTrials.gov (accessed on 12 December 2021) Identifier No.	Trial Title	Status
NCT03673124	GOG 3026: A Phase II Trial of Ribociclib Plus Letrozole in Women with Recurrent LGSC of the Ovary or Peritoneum	Recruiting
NCT03531645	A Pilot Phase II Study of Neoadjuvant Fulvestrant Plus Abemaciclib in Women With Advanced LGSC	Recruiting
NCT04575961	Phase II Investigational Study of Pembrolizumab Combination With Chemotherapy in Platinum-sensitive Recurrent Low-grade Serous Ovarian Cancer	Not yet recruiting
NCT04095364	A Randomized Phase III, Two-Arm Trial of Paclitaxel/Carboplatin/Maintenance Letrozole Versus Letrozole Monotherapy in Patients With Stage II-IV, Primary Low-Grade Serous Carcinoma of the Ovary or Peritoneum	Recruiting
NCT04625270	A Phase 2 Study of VS-6766 (Dual RAF/MEK Inhibitor) Alone and In Combination With Defactinib (FAK Inhibitor) in Recurrent Low-Grade Serous Ovarian Cancer	Recruiting
NCT05113368	Efficacy of Oral Regorafenib Combined With Intra-muscular Injection of Fulvestrant in Patients With Recurrent Low-grade Serous Ovarian Cancer: A Phase II Single Arm Trial	Not yet recruiting
NCT04625270	A Phase 2 Study of VS-6766 (Dual RAF/MEK Inhibitor) Alone and In Combination With Defactinib (FAK Inhibitor) in Recurrent Low-Grade Serous Ovarian Cance	Recruiting
NCT03909152	Basket Study of the Oral Progesterone Antagonist Onapristone ER (Apristor), Alone or In Combination With Anastrozole in Women With Progesterone Receptor Positive (PR+) Recurrent Granulosa Cell Tumor, Low Grade Serous Ovarian Cancer or Endometrioid Endometrial Cancer	Recruiting
NCTO4092270	A Study combining Peposertib (M3814) Pill with standard chemotherapy in patients with ovarian cancer with an expansion of high grade serous ovarian cancer and low grade serous ovarian cancer	Recruitment is suspended. Pending amendment
Not available	Molecular evaluation of MEK/ER response in LGSCs: A clinical Translational Study	Not yet recruiting
